# Deficiency of G1 regulators P53, P21^Cip1 ^and/or pRb decreases hepatocyte sensitivity to TGFβ cell cycle arrest

**DOI:** 10.1186/1471-2407-7-215

**Published:** 2007-11-19

**Authors:** Sharon Sheahan, Christopher O Bellamy, Donald R Dunbar, David J Harrison, Sandrine Prost

**Affiliations:** 1 Division of Pathology, Queen's Medical Research Institute, Edinburgh, UK; 2 Division of Pathology, Royal Infirmary of Edinburgh, Edinburgh, UK; 3 Bioinformatics, Queen's Medical Research Institute, Edinburgh, UK

## Abstract

**Background:**

TGFβ is critical to control hepatocyte proliferation by inducing G1-growth arrest through multiple pathways leading to inhibition of E2F transcription activity. The retinoblastoma protein pRb is a key controller of E2F activity and G1/S transition which can be inhibited in viral hepatitis. It is not known whether the impairment of pRb would alter the growth inhibitory potential of TGFβ in disease. We asked how *Rb*-deficiency would affect responses to TGFβ-induced cell cycle arrest.

**Results:**

Primary hepatocytes isolated from *Rb-floxed *mice were infected with an adenovirus expressing CRE-recombinase to delete the *Rb *gene. In control cells treatment with TGFβ prevented cells to enter S phase via decreased cMYC activity, activation of P16^INK4A ^and P21^Cip ^and reduction of E2F activity. In *Rb*-null hepatocytes, cMYC activity decreased slightly but P16^INK4A ^was not activated and the great majority of cells continued cycling. *Rb *is therefore central to TGFβ-induced cell cycle arrest in hepatocytes. However some *Rb*-null hepatocytes remained sensitive to TGFβ-induced cell cycle arrest. As these hepatocytes expressed very high levels of P21^Cip1 ^and P53 we investigated whether these proteins regulate pRb-independent signaling to cell cycle arrest by evaluating the consequences of disruption of *p53 *and *p21*^*Cip1*^. Hepatocytes deficient in *p53 or p21*^*Cip1 *^showed diminished growth inhibition by TGFβ. Double deficiency had a similar impact showing that in cells containing functional pRb; P21^Cip ^and P53 work through the same pathway to regulate G1/S in response to TGFβ. In *Rb*-deficient cells however, *p53 *but not *p21*^*Cip *^deficiency had an additive effect highlighting a pRb-independent-P53-dependent effector pathway of inhibition of E2F activity.

**Conclusion:**

The present results show that otherwise genetically normal hepatocytes with disabled *p53*, *p21*^*Cip1 *^or *Rb *genes respond less well to the antiproliferative effects of TGFβ. As the function of these critical cellular proteins can be impaired by common causes of chronic liver disease and HCC, including viral hepatitis B and C proteins, we suggest that disruption of pRb function, and to a lesser extend P21^Cip1 ^and P53 in hepatocytes may represent an additional new mechanism of escape from TGFβ-growth-inhibition in the inflammatory milieu of chronic liver disease and contribute to cancer development.

## Background

Transforming growth factor β (TGFβ) has characteristically diverse biological effects. Depending on the cell type and state of differentiation, TGFβ can either stimulate or inhibit proliferation, affect differentiation, promote extracellular matrix (ECM) formation and epithelial-to-mesenchymal transition (EMT), regulate cell adhesion, promote or inhibit cell migration and induce apoptosis (reviewed in reference [1]). In the liver, TGFβ is a critical mediator of multiple responses to injury [2]. Liver cell death in acute and chronic liver diseases is accompanied by inflammatory cell infiltration of the parenchyma and cytokine release including TGFβ. In such settings there develops an autocrine release of TGFβ from activated stellate cells, stimulating synthesis of ECM resulting in fibrosis [3]. At the same time, while hepatocytes and other intrinsic liver cells are stimulated to proliferate to compensate for cell loss, TGFβ is one of the signals that limit the proliferation of regenerating hepatocytes [4]. In advanced human liver cancer (hepatocellular carcinoma, HCC), there is commonly ectopic TGFβ production by the malignant hepatocytes in addition to that released by the non-parenchymal cells ([5,6]), giving rise to the idea that HCC cells have acquired a resistance to TGFβ-mediated growth inhibition. Current evidence suggests there is heterogeneity of resistance mechanisms that include decreased TGFβ-receptor II expression in early and late stage HCC [6] or induction of the inhibitory SMAD7 in advanced HCC [6-8]. These two adaptations have not been found in premalignant hepatocytes (dysplastic foci and regenerative nodules) in the chronically diseased liver, which are nevertheless similarly exposed to local TGFβ. It is reasonable to suppose that any resistance of pre-malignant hepatocytes to the anti-proliferative effects of TGFβ is likely to provide selective growth advantage in chronic liver disease favouring expansion into dysplastic nodules that are the precursor of HCC.

Studies in a variety of epithelial cells, including hepatocytes, have suggested at least two interconnected mechanisms by which TGFβ normally inhibits proliferation: downregulation of c-*myc *in early G1 and inhibition of cyclin-dependent kinase (CDK) activities by regulation of cyclin-dependent kinase inhibitors (CDKI) ([9] and therein) leading to the maintenance of pRb in the active hypophosphorylated form ([10]) and inhibition of S phase entry. These pathways point at critical proteins whose function is often altered during hepatocarcinogenesis, specifically the tumor suppressor pRb and P53 and the CDKI P21^Cip1 ^[10-13]. pRb, P53 and P21^Cip1^are critical regulators of the cell cycle that have all been involved in the antiproliferative effect of TGFβ in various systems. However, the fact that pRb phosphorylation is the main target of the regulation G1/S progression by P53 and P21^Cip1 ^makes it difficult to identify other possible pathways, independent of pRb.

Interestingly, both hepatitis B and hepatitis C viruses (HBV and HCV) express proteins that decrease expression or inhibit the function of pRb [14-16], P53 [17-19] and P21^Cip1 ^[20-22]. We hypothesised that such dysfunctional pRb, P53 and P21^Cip1 ^in chronic liver disease reduce the growth inhibitory response of affected hepatocytes to the TGFβ-rich environment of cirrhosis [5,6]. Using primary murine hepatocytes deficient in these genes singly or in combination we sought to determine firstly whether there was a loss of sensitivity to TGFβ-mediated cell cycle arrest and apoptosis and also to determine the relative contribution from each of *p53*, *p21*^*Cip1 *^and *Rb*-deficiency.

## Methods

### Hepatocyte culture

Mouse primary hepatocytes (male, 6–12 weeks old), were isolated by a standard two-steps retrograde perfusion procedure [23] and purified using percoll gradient [24]. The obtained hepatocytes were cultured in supplemented serum-free medium selecting against survival of non-parenchimal cells [25,26]. Where appropriate, hepatocytes cultured for 24 hours were treated daily with 160 pM of TGF-β1 (TGFβ) for the indicated time.

Mice were produced by crossing *p53*-/- [27] with *Rb*-floxed mice (homozygous for exon 19 of *Rb *flanked by LoxP sequences) [28] and *p21*^*Cip1*^-null mice (*p21-/-*) [29] as previously described [30]. *Rb*-deficient isogenic cells were obtained by deletion of the *Rb*-floxed alleles *in vitro *by infection with an adenovirus expressing Cre-recombinase (Ad-Cre) using a multiplicity of infection of 10 [30]. Control cells, infected with a replication-deficient adenovirus (Ad-Dl70) are phenotypically wild-type. All animals used in this study received humane care. The study protocols are in compliance with the UK Home Office regulation and the local institutional policies.

### Proliferation

In the present isolation and culture conditions, isolated hepatocytes are more or less synchronous, with the majority of cycling wild type cells entering S phase 72 hours after plating and going into mitosis around 96 hours after plating [25,31]. As TGFβ inhibits proliferation via G1 block, we assessed changes in proliferation by quantifying the number of hepatocytes undergoing replicative DNA synthesis (S phase) by immunodetection of BrdU incorporation. Briefly, hepatocytes were incubated with 40 μM BrdU for 6 hours and fixed in 80% ethanol. Immunodetection was performed using Rat anti-BrdU IgG (SeraLabs, Sussex) 1/100 dilution and rabbit anti-rat IgG HRP-conjugate 1/100 dilution. Slides were counter-stained with haematoxylin and light-green. Negative controls omitted BrdU.

To compare the effect of TGFβ in hepatocytes of different genotypes, we calculated the percentage of inhibition of proliferation by TGFβ for 2 (for *p53p21-/- *and TRPL) to 6 independent experiments: proliferation was integrated between 48 and 96 hours after plating (i.e. cells treated or not with TGFβ for 24 to 72 hrs) using Kaleidagraph (SynergySoftware) giving the "mean proliferation" between these time points. The inhibition of proliferation was calculated as 100-(100*(mean proliferation of TGFβ-treated cells/mean proliferation of untreated cells)).

### Immunofluorescence

Hepatocytes were fixed in acetone/methanol (1:1 v/v). Immunodetection was performed using anti-P53 mouse monoclonal antibody (1:1000) (AB-1, OncogeneScience UK), rat anti-P16^INK4A ^polyclonal (AB3004, Chemicon), monoclonal mouse anti-human-P21^Cip1 ^(SX118, Dako), mouse monoclonal anti-P27^KIP1 ^(BD-Pharmingen), the appropriate Alexafluor (Molecular Probes) secondary antibody (1/200), followed by Topro-3 nuclear counterstain. Quantification was performed by manual counting on 25 representative fields (×40) photographed with a Zeiss confocal microscope. Scanning was performed using multitracking, and settings constant throughout the experiments.

### E2F and MYC transcriptional activity

Hepatocytes in culture for 48 hours were transfected using TFx-50 (Promega) reagent (ratio 1/5 w/w DNA/lipid) [32]) with p-TA-*Luc *(control reporter), p-*E2F*-TA-*Luc *(*E2F *reporter), or p-*myc*-TA-*Luc *(*c-myc *reporter) (all from Pathway Profiling System4, Clontech). All drive the firefly luciferase gene (*Luc*) (for detailed map see [33]). Thirty hours after transfection, luciferase activity was quantified using Luciferase Assay reagents according to the manufacturer's instructions (Promega) and corrected for the quantity of protein (Biorad assay). The data are given relative to the expression in untreated control cells.

### Gene expression

Total RNA was isolated at indicated times using QIAGEN RNeasy mini Kit according to the manufacturer's instructions. RNA quality was determined with a Bioanalyzer (RNA6000 NanoLabChip kit, Agilent 2100 Bioanalyzer, USA). Expression analysis was performed using the GEArrayQ Series Mouse Cell Cycle Kit, (Superarray, USA) where each gene is represented by 4 independent spots. The cDNA was prepared from total RNA using Superarray AmpoLabeling-LPR Kit, USA and labelled with Biotin-16-dUTP (Rocha).

Images of the arrays were obtained using a Versadoc detector (BioRad, UK) and converted into raw data using Scanlyzer (Michael Eisen, Lawrence Berkely Bation Lab, USA). The data was analysed using the GEArrayAnalzer Software (Version 1.0) with background subtraction (using plasmid DNA PUC18 as negative control) and normalisation with the housekeeping gene Ppia (cyclophlinA). The normalization removes differing intensity scales from the experimental readings, allowing comparison between experiments.

### Statistical analyses

Data and statistical analyses were done with Minitab 13.0 and Spotfire Decision site softwares. The proportion of affected cells was arcsine transformed to normalise the distribution, and differences between means were evaluated with Analysis of Variance (ANOVA). Differences were taken to be significant when p < 0.05. Satisfactory homogeneity of variances was determined with Bartlett's test. Where a significant difference between means was identified with ANOVA, the differences between individual means were analysed further with Bonferroni simultaneous tests for multiple comparisons.

## Results

pRb and other pocket proteins are central to the regulation of G1/S transition by inhibition of E2F activity and transcription of multiple target genes involved in DNA synthesis and cell cycle regulation. In *Rb*-deficient hepatocytes, although E2F activity is elevated [30], we found using gene expression array (see methods) that expression of *p107 *and *p130 *were increased (2.1, 2 and 3.1 fold for *p107*; 1.3, 1.1 and 1.4 fold for *p130 *at 48, 72 and 96 hours after plating). This may help maintain some regulation of G1/S transition including inhibition of proliferation by TGFβ. Studies in a variety of epithelial cells, including hepatocytes, have shown at least two interconnected mechanisms by which TGFβ normally inhibits proliferation: downregulation of *c-myc *in early G1 and inhibition of cyclin-dependent kinase (CDK) activities by regulation of cyclin-dependent kinase inhibitors (CDKI) ([9] and therein) leading to the maintenance of pRb, and other pocket proteins in the active hypophosphorylated form [10] and inhibition of E2F responsive promoters. We asked what would be the consequences of *Rb *deletion on TGFβ regulation of hepatocytes proliferation.

**Figure 1:**
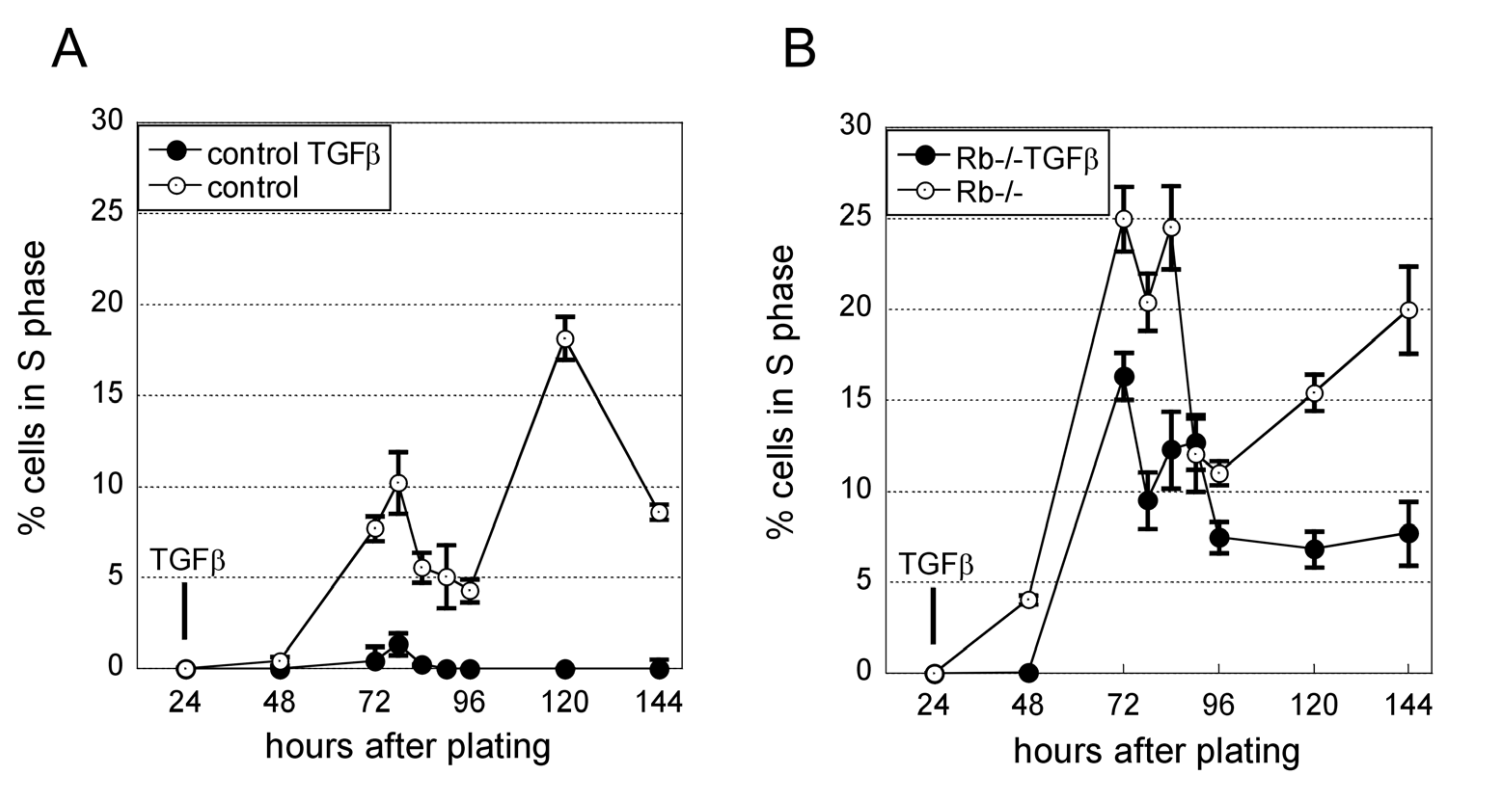
***Rb *deficiency reduces TGFβ inhibition of proliferation**. The figure shows the percentage of cells in S phase at the indicated times after plating of one representative experiment. All hepatocytes for the experiment were isolated from the same *Rb*-floxed mouse. Control and *Rb*-/- cells are *Rb*-floxed cells treated at the time of plating with either adenovirus control (wild-type phenotype) (A); or adenovirus expressing Cre (*Rb*-/-) (B) respectively. Each point is the average proliferation in 2 independent cultures where 500 hepatocytes were counted +/- SEM. The experiment was repeated multiple times with similar results. Where appropriate (close symbols), TGFβ was added daily from 24 hours after plating.

In our culture conditions, primary hepatocytes enter S phase in a more or less synchronous manner. The first control cells reach S phase around 72 hours after plating (Figure 1A) and M phase is observed around 96 hours [25,30,31]. Hepatocytes can enter a second cell cycle but often in a less synchronous manner. As we have previously reported [30] following *Rb *deletion within the first 24 hours after plating a higher number of cells enter S phase and there is an earlier onset of DNA synthesis which is detected as soon as 48 hours after plating (Figure 1A & B compare curves with open symbols).

### *Rb *is central to TGFβ-induced inhibition of proliferation

In these conditions, TGFβ-treatment of control cells was highly effective to cause growth arrest almost completely blocking proliferation (Figure 1A). By contrast, many of the sister cells subjected to inducible *Rb*-deletion escaped the inhibition by TGFβ and entered S phase (Figure 1B).

**Figure 2:**
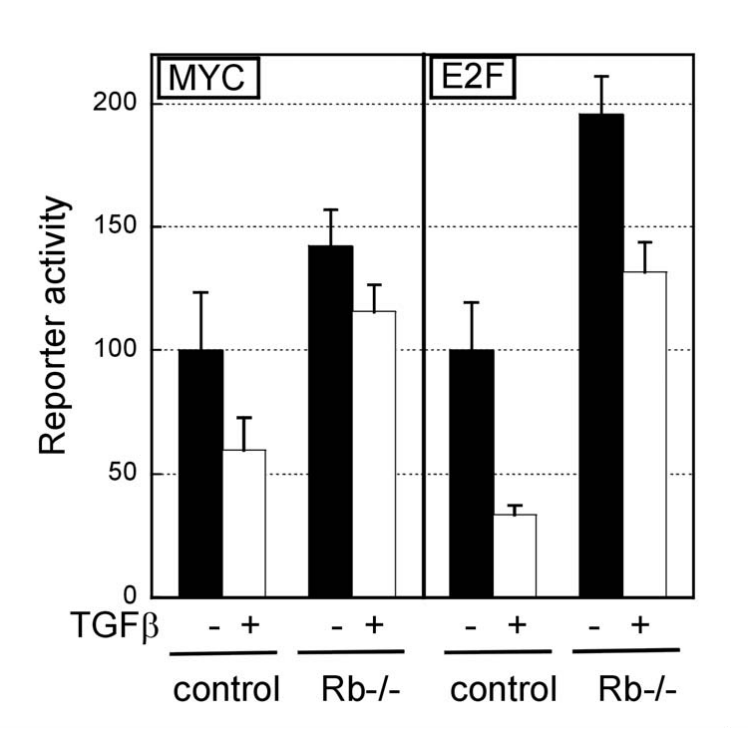
**TGFβ affects c-MYC and E2F transcriptional activity**. The graph represents the transcriptional activities of c-MYC and E2F quantified using a reporter assay. Control and *Rb*-/- hepatocytes were in culture for 78 hours, treated or not with TGFβ for 30 hours at the time of the assay. The bars represent the average +/- SEM of duplicate transfections.

E2F and MYC activities were found to be significantly higher in *Rb*-null cells compared with wild type (Figure 2, compare black bars). This was detectable from 48 hours for E2F (data not shown) and 72 hours after plating for MYC (Figure 2). TGFβ-treatment decreased MYC activity in both control and *Rb*-null hepatocytes, although this was less efficient in *Rb*-null cells (40.3% and 18.5% decrease for control and *Rb*-null respectively) and the activity remained higher than in untreated control hepatocytes (Figure 2). E2F activity also decreased after TGFβ-treatment in cells of either genotype (32.6 in *Rb*-/- and 66.4% in control).

**Figure 3:**
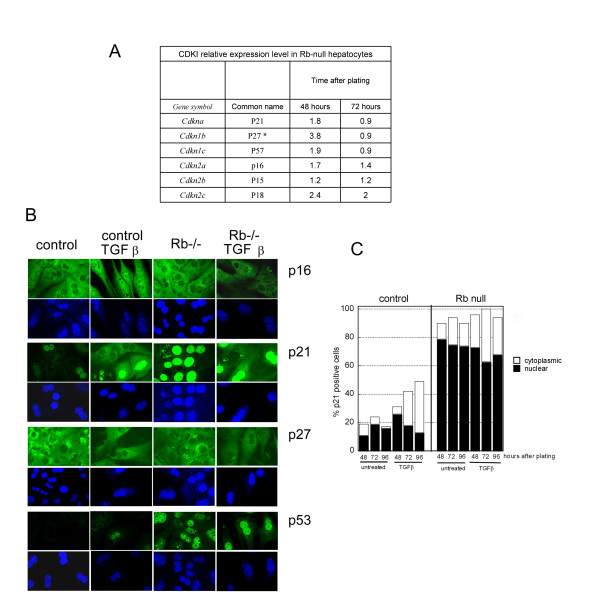
**Effect of TGFβ on P53, P16^INK4A^, P21^Cip1 ^and P27^KIP1 ^expression** A: Expression of CDKI in *Rb*-null hepatocytes. The table gives the level of expression of the various CDKI in *Rb*-null hepatocytes relative to control hepatocytes. The values were obtained using a gene expression array as described in methods. * The level of p27^KIP1 ^expression in control cells was low, and the ratio may therefore be overestimated. B & C. Hepatocytes were treated or not with TGFβ 24 hours after plating. B. Immunofluorescence for P16^INK4A^, P21^Cip1^, P27^KIP1 ^and P53. Photos were taken 48 hours after treatment. Green: specific immunofluorescence, blue: Topro-3 nuclear counterstain. C: Quantification of P21^Cip1 ^immunopositivity. Black bars: percentage of cells exhibiting nuclear staining. White bars, cytoplasmic staining.

MYC is a negative regulator of CDKI expression [34-37]. Accordingly, we found that the high levels of CDKI expression initially observed in response to *Rb *deletion (48 after plating), returned to wild type levels at 72 hours, together with the increased MYC activity (Figure 2, Figure 3A). We can only speculate about the mechanism behind the initial increase of CDKI expression, but the suggestion that E2F activity can regulate CDKI expression [38] suggests that this may be an early response to the rapid increase of E2F activity resulting from the induced *Rb *deletion, before the system reaches an equilibrium.

Decreased MYC expression, as observed here in response to TGFβ is known to alleviate inhibition of transcription of various CDKI. We therefore investigated CDKI expression in response to TGFβ. We were unable to detect P15^INK4B ^in hepatocytes of either genotype; P27^KIP1 ^expression was similar in control and *Rb*-null cells and was not affected by TGFβ (Figure 3B). P16^INK4A ^appeared to localise in the nuclei after TGFβ-treatment of control but not *Rb*-null cells (Figure 3B). P16^INK4A ^inhibits CDK4 and CDK6 that are known to phosphorylate pockets proteins. P16^INK4A ^may therefore contribute to TGFβ-induced cell cycle arrest by inhibition of pocket protein phosphorylation and reduction of E2F activity in control but not in *Rb*-null cells. In control cells TGFβ-treatment was also accompanied by early activation of P21^Cip1 ^(Figure 3B & C) which relocalised in the cytoplasm: the percentage of nuclear positivity increased initially to rapidly drop back to the level of untreated cells but with a concomitant increase of cytoplasmic P21^Cip1^-staining (Figure 3B, 3C) from 72 hours after plating. In *Rb*-null cells, which show a constitutive high level of P21^Cip1 ^due to P53 activation [30], there was no change in nuclear positivity but an increased number of cells exhibited cytoplasmic staining (Figure 3B, 3C). Finally, TGFβ marginally affected P53, as few strongly positive cells were observed in TGFβ-treated control cells (<2%) and the small proportion of "less positive"*Rb*-null hepatocytes became intensively fluorescent with TGFβ-treatment (Figure 3B).

### Some *Rb*-deficient hepatocytes remain sensitive to TGFβ-induced inhibition of proliferation through a P53-dependent pathway

Interestingly, a certain proportion of *Rb*-/- hepatocytes were nevertheless prevented to by TGFβ from entering S phase (Figure 1B) as the number of cells incorporating BrdU is reduced (Figure 1B compare curves with open and black symbols).

As *Rb*-null greatly differed from control hepatocytes in respect of the high level of P21^Cip1^and P53, we investigated whether these proteins could contribute to TGFβ-induced cell cycle arrest independently of pRb. To address this question, we compared the inhibition of proliferation in hepatocytes knocked-out for these genes, undergoing or not a further inducible deletion of the *Rb *gene.

**Figure 4:**
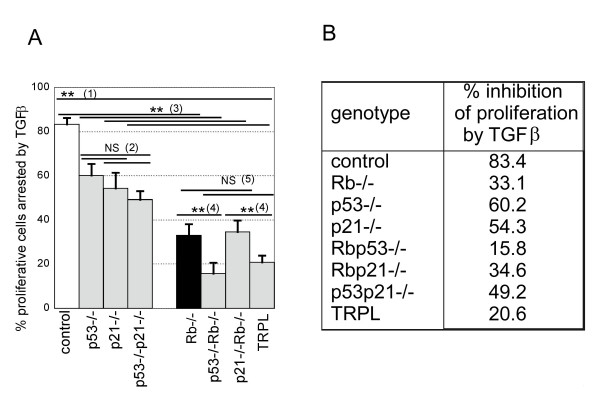
***Rb *is central to TGFβ inhibition of proliferation via multiple pathways**. A. The figure shows the effect of *p53*, *p21*^*Cip1 *^or *Rb *deficiency on TGFβ induced cell cycle arrest +/- SDV. The percentage inhibition of proliferation by TGFβ was calculated for 2 (for *p53p21-/- *and TRPL) to 6 independent experiments and differences analysed by ANOVA (** p < 0.0001; NS non significant). As for all experiments the *Rb*-null hepatocytes were obtained by infection at the time of plating of the *Rb-floxed *hepatocytes of corresponding genotypes with adenovirus expressing Cre recombinase. (1) all deficient hepatocytes respond less well to inhibition of proliferation by TGFβ than control cells and *Rb *deficient has the strongest effect. (2) *p53 *and *p21*^*Cip1*^deficiency, singly or together have a similar effect on inhibition of proliferation by TGFβ. (3) *Rb *deletion significantly reduces TGFβ-induced cell cycle arrest regardless of *p53 *and *p21*^*Cip1*^status (compare control with *Rb-/-*; *p53-/*- with *p53-/-Rb-/-; p21-/- *with *p21-/-Rb-/*- and *p53-/-p21-/- *with TRPL). (4) double deficiency in *Rb *and *p53 *further decreases hepatocytes responses to TGFβ in term of regulation of proliferation, independently of *p21*^*Cip1*^status. (5) by contrast the effect of TGFβ on hepatocytes deficient in both *p21*^*Cip1 *^and *Rb *is not significantly different to that of *Rb *null cells, and this is independent of *p53 *status. B. corresponding percentages of inhibition of proliferation for each genotype. TRPL: triple null hepatocytes.

*p21*^*Cip1 *^and *p53 *deficiencies similarly affected cell responses to TGFβ with about 55–60% of the proliferating cells being inhibited by TGFβ (Figure 4 legend (2)). Simultaneous deficiencies in both *p21*^*Cip1 *^and *p53 *did not significantly differ from either alone (Figure 4 legend (2)). As expected *Rb*-deficiency had the strongest effect, sharply decreasing the susceptibility to TGFβ-induced cell cycle arrest regardless of *p53 *and *p21*^*Cip1 *^genotypes (Figure 4 legend (3) all p < 0.0001). This confirms the central role played by pRb in the response to TGFβ.

**Figure 5:**
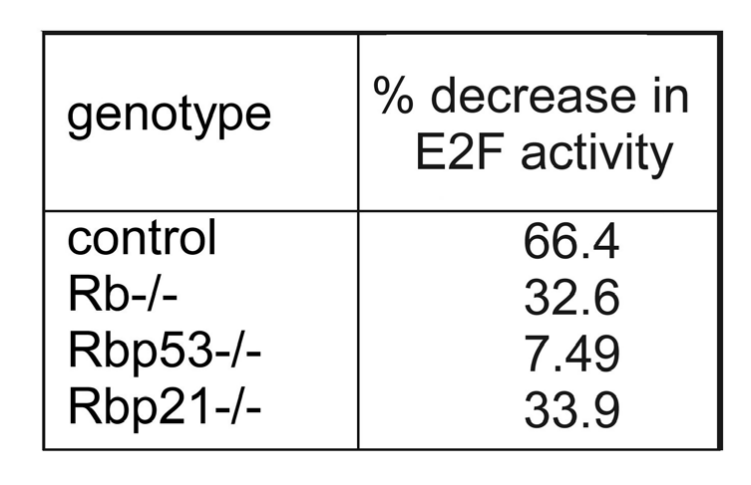
**Deficiency in *p53 *but not *p21*^*Cip1 *^further decreases E2F activity in *Rb*-deficient hepatocytes**. The values are the percentage decrease in E2F activity in TGFβ-treated hepatocytes of different genotypes compared with untreated cells. The E2F activity was quantified using a reporter assay as described in figure 2.

In *Rb*-null cells additional loss of *p53 *(Figure 4 legend (4)) but not *p21*^Cip1 ^caused a further reduction in sensitivity to TGFβ (*Rb*-/- versus *Rb-/-p53*-/-; p = 0.0001 and *Rb*-/- versus *p21*-/-*Rb*-/- p = 0.20). This was accompanied by a reduced inhibition of E2F activity by TGFβ in *Rb-/- p53-/- *but not in *Rb-/-p21-/- *(7.49% reduction in *Rb-/- p53-/- *and 33.9 in *Rb-/-p21-/- *versus 32.6 in *Rb-/- *hepatocytes) (Figure 5).

## Discussion

We have investigated here the consequences of *Rb*-deficiency in hepatocytes – as happens in viral liver diseases – with respect to TGFβ regulation of proliferation. Our data show that pRb is central to the anti-proliferative action of TGFβ with the majority of *Rb*-null cells escaping from this effect. This may have consequences for the development of cancer. We found however that a proportion of *Rb*-null hepatocytes remain sensitive to TGFβ and undergo cell cycle arrest and investigated whether P53 and P21^Cip1^could be involved.

TGFβ is a potent inhibitor of cell proliferation by activation of a cell cycle arrest in G1 through potentially multiple pathways: First, protein complexes containing SMAD3 [39-43] and P107 [44,45] downregulate MYC activity leading to CDKI upregulation ([1] and therein). The active CDKs that can feedback on SMAD3 to reduce its activity [46,47], are therefore kept in check by the CDKIs. Consistent with the involvement of P107 rather than pRb, we observed decreased MYC activity in both wild-type and *Rb*-null hepatocytes, albeit with somewhat reduced efficiency in the later. In the absence of P16^INK4A ^activation, SMAD3 may be inhibited by CDKs thus preventing optimum inhibition of MYC [39,40] in *Rb*-deficient cells.

A reported effect of c-*myc *downregulation is to reduce P15^INK4B ^repression and inhibit cyclinD/CDK4 and cyclinE/CDK2 by both direct binding and relocalisation of P27^KIP1 ^from cyclinD/CDK4 to cyclinE/CDK2 [1,48]. Although in untreated *Rb*-null hepatocytes, all CDKI are overexpressed in G1, we were unable to detect expression of P15^INK4B ^and did not observe any changes in P27^KIP1 ^after TGFβ-treatment. P16^INK4A ^which localised in the nuclei of control but not *Rb*-null cells after TGFβ-treatment can inhibit CDK4 & 6 and may therefore contribute to TGFβ-induced cell cycle arrest by inhibition of pocket protein phosphorylation and reduction of E2F activity in control but not in *Rb*-null cells.

Downregulation of c-*myc *also allows activation of *p21*^*Cip1*^[49], and our finding that TGFβ-treatment greatly increased nuclear P21^Cip1 ^could certainly contribute to cell cycle arrest via inhibition of pRb phosphorylation in cells containing *Rb*.

By contrast, it is interesting that *p21*^*Cip1 *^deficiency in *Rb*-null hepatocytes did not reduce the sensitivity of hepatocytes to TGFβ (correlated with a similar decrease in level of E2F activity; 32.6 and 33.9% in *Rb*-/- and *Rbp21-/*- respectively). We have indeed previously shown that P21^Cip1 ^provides pRb-independent control of hepatocytes proliferation: in standard culture conditions *p21-/-Rb-/*- hepatocytes proliferate more than hepatocytes bearing only one knock-out gene [30]. Various potential mechanisms have been discussed and include the inhibition of PCNA [50], or cyclin E/cdk2 [51,52] or reduction of MYC activity [53] by P21^Cip1^. The present results therefore suggest that whatever the mechanism involved, it is not enhanced by TGFβ-treatment and that proliferation rate and inhibition of proliferation by TGFβ are unrelated.

Thus activation of cyclin kinase inhibitors P21^Cip1 ^and P16^INK4A ^can therefore inhibit pRb and other pocket proteins phosphorylation and prevent E2F transcriptional activity leading to very efficient cells cycle arrest in control cells.

In *Rb*-null hepatocytes where neither CDKI seem involved after TGFβ-treatment, E2F activity was nevertheless reduced suggesting that a different mechanism may affect other pocket protein(s) and contribute to cell cycle arrest of *Rb*-null hepatocytes. P21^Cip1 ^and P53 were both strongly increased in *Rb*-null hepatocytes and shown to change with TGFβ treatment; we therefore investigated if they could contribute to TGFβ-induced inhibition of proliferation through an *Rb*-independent pathway.

By comparing proliferative responses of hepatocytes of various genotypes, a P53-dependent, P21^Cip1^-independent pathway was highlighted. This was correlated with a greater reduction of E2F activity in *Rb-/-p53-/*- cells suggesting that high P53 prevents inhibition of E2F activity by TGFβ. This may involve P53-dependent inhibition of CDK4 expression – indeed we find that in *p53-/*- hepatocytes CDK4 expression in G1 and S phase is reduced to 76% and 72% of the level in control cells – or repression of CDK4 synthesis [54] and prevent E2F release from P107 which we have shown to be twofold increased in *Rb*-null cells.

## Conclusion

Loss of responsiveness to TGFβ antiproliferative effects is believed to be important in carcinogenesis, yet the known mechanisms of TGFβ resistance happen late in the progression of established liver cancer [55,8]. The present results show that otherwise genetically normal hepatocytes with disabled *p53*, *p21*^*Cip1 *^or *Rb *genes respond less well (by differing degrees) to the antiproliferative effects of TGFβ. As the function of these critical cellular proteins can be impaired by common causes of chronic liver disease and HCC, including viral hepatitis B and C proteins [14-22], we suggest that disruption of pRb function, and to a lesser extend P21^Cip1 ^and P53 in hepatocytes may represent an additional new mechanism of escape from TGFβ growth inhibition in the inflammatory milieu of chronic liver disease.

## Abbreviations

Transforming growth factor β: TGFβ; 5-bromo-2-deoxyuridine: BrdU; Standard error of the mean: SEM; hepatocellular carcinoma: HCC; HCV: hepatitis C virus; HBV hepatitis B virus; CDK: cyclin-dependent kinase; CDKI: cyclin-dependent kinase inhibitor.

## Competing interests

The author(s) declare that they have no competing interests.

## Authors' contributions

SS carried initiated the study and carried out some of the experiments, COB contributed to the experimental design, interpretation of data, supervision and gave critical review of the manuscript, DRD helped with the interpretation of data using Spotfire Decision site software, DJH contributed to the experimental design and gave general supervision and funding support, SP made substantial contribution to the conception & design, acquisition and interpretation of data and wrote the manuscript.

## Pre-publication history

The pre-publication history for this paper can be accessed here:


